# Industry affiliation influence on randomized controlled trials for platelet-rich plasma in the treatment of lateral epicondylitis: a systematic review

**DOI:** 10.1016/j.jseint.2024.06.010

**Published:** 2024-07-04

**Authors:** Justin B. Castonguay, Jacob L. Kotlier, Amir Fathi, Frank A. Petrigliano, Joseph N. Liu

**Affiliations:** a University of Illinois College of Medicine, Chicago, IL, USA; b University of Southern California Keck School of Medicine, Los Angeles, CA, USA; c Department of Orthopaedic Surgery, University of Southern California Keck School of Medicine Los Angeles, Los Angeles, CA, USA

**Keywords:** Platelet-rich plasma, Lateral epicondylitis, Tennis elbow, PRP, Industry affiliation, Extensor carpi radialis brevis

## Abstract

**Background:**

Explicit funding and industry affiliation are believed to potentially impact medical research. There have been an increasing number of studies that have evaluated this relationship. The purpose of this study is to determine whether industry affiliation influences the outcomes of randomized controlled trials that investigate the effectiveness of platelet-rich plasma (PRP) in the treatment of lateral epicondylitis.

**Methods:**

A search of PubMed, SPORTDiscus, and SCOPUS was performed using the search terms “lateral epicondylitis” and “platelet-rich plasma” as well as “tennis elbow” and “platelet-rich plasma.” Only studies from 2010 to present were considered. Final texts were then analyzed for industry affiliation and treatment efficacy. When determining whether a study was industry-affiliated, explicit financial supporters of the study, stated conflicts of interest, American Academy of Orthopaedic Surgeons disclosures, and the Centers for Medicare and Medicaid Services open payments database were assessed. Study outcomes were categorized as favorable, analogous, or unfavorable based on achieving a statistically significant (*P* < .05) comparison between PRP and control.

**Results:**

A total of 26 studies were used. Of these, 20 were industry nonaffiliated and 6 were industry affiliated. There were 15 studies (2 affiliated and 13 nonaffiliated) that reported PRP as favorable compared to the comparison (corticosteroid, analogous whole blood, or normal saline). The endpoints were 6 and 12 months after the use of PRP or the comparison. Quantitative data analysis yielded results that were not statistically significant between industry-nonaffiliated and affiliated groups. The *P* values for 6-month visual analog scale, 12-month visual analog scale, 6-month disabilities of the arm, shoulder, and hand, 12-month disabilities of the arm, shoulder, and hand, 6-month patient-related tennis elbow evaluation, and 12-month patient-related tennis elbow evaluation were 0.577, 0.635, 0.554, 0.465, 0.273, and 0.157, respectively.

**Conclusion:**

Despite our results indicating that industry affiliation does not have an impact on outcomes of randomized controlled trials examining the treatment of lateral epicondylitis with PRP, it is important for future studies to evaluate affiliations when making treatment recommendations.

While there have been other studies that have investigated the influence of industry involvement on the outcomes of trials assessing the efficacy of platelet-rich plasma (PRP) for the treatment of other orthopedic conditions,^41^ there have been no prior studies investigating this with the treatment of lateral epicondylitis.

Biologics such as PRP have been increasingly explored for various conditions in the field of orthopedics. There have been numerous studies investigating its efficacy in the treatment of conditions such as rotator cuff tears, knee osteoarthritis, Achilles ruptures and tendinopathy, and meniscal repairs.^19^ There have been an increasing amount of studies investigating the efficacy of PRP in the treatment of lateral epicondylitis, particularly how it compares to corticosteroid injection.^14^ Common patient-reported outcome measures (PROMs) that these studies use include the visual analog scale (VAS), disabilities of the arm, shoulder, and hand (DASH), and patient-related tennis elbow evaluation (PRTEE).^47^^,^^15^ One review investigating 26 studies determined that PRP provides improvements in VAS, DASH, and PRTEE starting at 4 weeks and VAS and DASH at 8 weeks for PRTEE.^39^

Studies in orthopedics and other medical fields have investigated how industry affiliation and explicit funding influence the outcomes of randomized controlled trials (RCTs). One review demonstrated that studies with industry affiliation and explicitly stated conflicts of interest have a statistically significant greater likelihood of reporting positive outcomes results relative to nonaffiliated studies.^40^ Other studies have reported similar findings across both orthopedic and other medical literatures.^30^^,^^22^^,^^34^^,^^45^ One orthopedic review investigated 100 orthopedic clinical trials across several journals found that 22/26 industry affiliated trials reported favorable outcomes and were 10.9 times more likely to report favorable findings compared to nonaffiliated studies.^17^ However, other orthopedic reviews did not find an association between industry affiliation and more favorable outcomes.^41^^,^^23^^,^^9^ A study by Foughty et al investigated 244 articles with randomized control trials in the Journal of Shoulder and Elbow Surgery, and after data analysis, their results demonstrated that articles with conflicts of interest were not more likely to have positive outcomes compared with articles without conflicts of interest.^9^

The purpose of this study is to characterize the effect of industry affiliation on study outcomes in RCTs investigating PRP use in lateral epicondylitis. To our knowledge, no previous studies have investigated this question.

## Materials and methods

One author (J.C.) queried PubMed, SPORTDiscus, and Scopus using search terms “lateral epicondylitis” and “platelet-rich plasma”; “tennis elbow” and “platelet-rich plasma” filtering for results from 2010 to present was conducted by two independent authors (J.K. and J.C.). Inclusion criteria were: Level 1 or Level 2 randomized control trials comparing PRP vs. other strategies in the treatment of elbow epicondylitis. Exclusion criteria were: animal studies, cadaver studies, systematic reviews, meta-analyses, case studies, case reviews, studies with no PROMs (VAS, DASH, or PRTEE), no comparison treatment, and not level 1 or level 2 studies. Only studies in English and those within a timeframe of 2010 to present were used. Full-text screening was done by two authors (J.K. and J.C.) with discrepancies resolved by a third (A.F.). All authors have adhered to the Preferred Reporting Items for Systemic Reviews and Meta-Analysis guidelines. No ethical approval was required.

When determining which studies to include, an analysis of potential bias in each study was conducted. In order to do this, we utilized the Cochrane Handbook for Systematic Review of Interventions. The exclusion criteria that were established were done so to minimize the selection of studies that may exhibit bias. Only RCTs that had either level 1 or level 2 evidence were used in this systematic review to prevent the methodology of the studies from impacting the results. As a way of avoiding attrition bias and reporting bias, only studies with complete outcome data were used.

Once studies had been identified, industry affiliation was determined by assessing the conflict of interest (COI), funding, and disclosure sections of included publications. All study authors were also assessed through the American Academy of Orthopaedic Surgeons (AAOS) and the Centers for Medicare and Medicaid Service (CMS) open payments database (both were last searched on January 16, 2024). Both AAOS and CMS were used to determine industry affiliation because the CMS open payments database does not report on non-American authors. The criteria for industry affiliation were Medical Degree and Doctor of Osteopathic Medicine physicians that disclosed affiliation to a company that occurred in the years prior to submission of the author’s RCT results. Disclosures made after the submission were not included.

COI and funding were identified by explicitly stated sponsorship from PRP administration device manufacturers such as Zimmer Biomet, Arthrex, Stryker, and others. These studies were deemed to be industry-affiliated for data analysis. Studies that received funding from nonindustry-affiliated sources, such as the local hospital, were counted as nonaffiliated in the data analysis.

The outcomes of each study were rated as favorable, analogous, or unfavorable for each patient-reported outcome at 6-month and 12-month timepoints. Studies were deemed favorable if they reported a statistically significant improvement in patient-reported outcomes with the use of PRP compared to corticosteroids or normal saline at 6-month and/or 12-month timepoints. Studies deemed analogous had no statistically significant change at those timepoints. Studies deemed unfavorable had statistically significant worse patient outcomes with PRP.

When reviewing the 26 studies that met the inclusion criteria, there were various timepoints reported ranging from 3 weeks to 2 years. The timepoints for VAS, DASH, and PRTEE in our study’s analysis were 6-month and 12-month due to those being the most common two reported timepoints.

Statistical analysis was performed using Microsoft Excel (Version 16.77; Microsoft Corp., Redmond, WA, USA). The data were reported in a descriptive fashion. Patient-reported outcomes (VAS, DASH, and PRTEE) were compared between industry-affiliated and nonindustry-affiliated studies using chi-square statistical analysis. Studies were deemed to be statistically significant if the chi-square test yielded a *P* value < .05.

## Results

**Figure 1:**
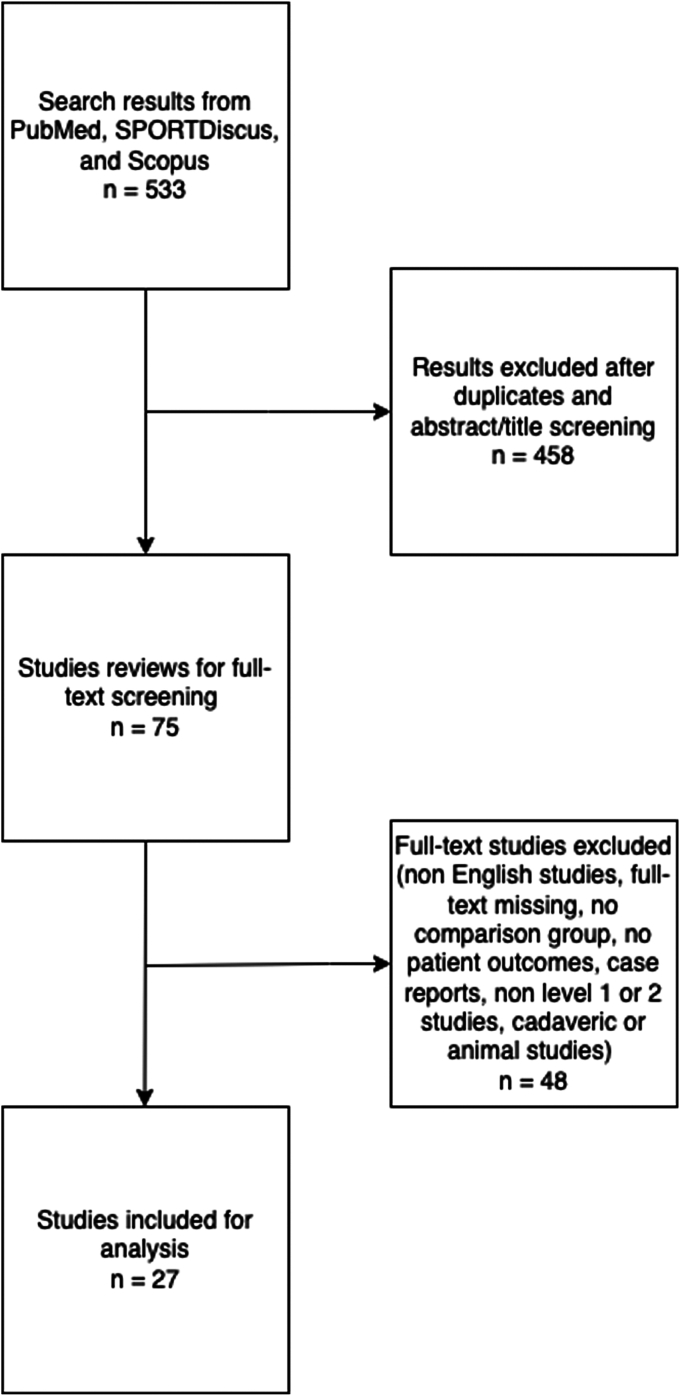
Inclusion criteria in the PRISMA flowsheet. This is a summary of the exclusion criteria listed in the manuscript. It shows that initially there were 533 studies from PubMed, SPORTDiscus, and Scopus. After duplicates were removed, abstract/title screening, full-text screening, and exclusion criteria, it shows the total studies used for analysis. *PRISMA*, Preferred Reporting Items for Systemic Reviews and Meta-Analysis; *PRP*, platelet-rich plasma; *RCT*, randomized controlled trial.

Initially, our search resulted in 533 possible results from PubMed, SPORTDiscus, and Scopus. After removing duplicates, studies by title, and completing abstract screening, there were 75 possible results. Two independent authors conducted a full-text screening, which resulted in 26 studies that met inclusion criteria and were used in our data analysis. Preferred Reporting Items for Systemic Reviews and Meta-Analysis flow diagram can be found in Figure 1.

There were a total of 6 (23.1%) industry-affiliated studies and 20 (76.9%) nonaffiliated studies that were analyzed (Table 1). All studies were either level 1 (73.1%) or level 2 (26.9%). A complete summary of included studies, including the level of evidence, explicit financial supporters of the study, COI/funding section indicating affiliation, AAOS affiliation, and CMS affiliation can be found in Table 2.

**Table I:** Quantitative data of affiliated vs. nonaffiliated.

Outcome	Affiliated (n = 6)	Nonaffiliated (n = 20)	*P* value
Favorable	2 (33.33%)	13 (65%)	.166
Analogous	4 (66.67%)	7 (35%)	.169

**Table II:** Included studies.

Author	Level of evidence (1 or 2)	PRP/PRP administration device manufacture(s) in study	Explicit financial supporters of study	COI/funding section demonstrates affiliation (Y/N)	AAOS author demonstrates affiliation (Y/N) https://disclosure.aaos.org/	CMS author search demonstrates affiliation (Y/N) https://openpaymentsdata.cms.gov/
Seetharamaiah^39^	1	None listed	None	None	None	None
Lebiedzinski^20^	1	Double Syringe System, Arthrex	None	None	None	None
Watts^44^	1	Zimmer Biomet	Yes (Zimmer Biomet)	None	Yes (Watts, Arthrex, Biomet, Stryker)	None
Yerlikaya et al^47^	1	None listed	None	None	None	None
Yadav^46^	1	None listed	None	None	None	None
Sahbaz^38^	2	None listed	None	None	None	None
Gungor^12^	2	None listed	None	None	None	None
Raeissadat^35^	1	Arya Mabna Tashkis Corporation, RN: 312569)	None	None	None	None
Khaliq^16^	1	None listed	None	None	None	None
Palacio^32^	1	None listed	None	None	None	None
Mishra^27^	2	Biomet	Yes (Biomet)	Yes (Biomet)	Yes (Mishra, DePuy, Zimmer)	Yes (Mishra, Arteriocyte Medical Systems, Zimmer, DePuy)
Creaney^5^	1	None listed	None	None	None	None
Montalvan^28^	1	Arthrex	Yes (Arthrex)	None	Yes (Philippe Hardy, Arthrex, Ceraver Osteal, OTSR, Zimmer)	None
Raeissadat^36^	1	Arya Mabna Tashkis Corporation, RN: 312569)	None	None	None	None
Kamble^15^	2	None listed	None	None	None	None
Behera^3^	2	None listed	None	None	None	None
Omar^31^	1	JMS Singapore Ltd.	None	None	None	None
Arora^2^	1	None listed	None	None	None	None
Gosens^11^	1	Biomet Biologics	Yes (Biomet)	Yes (Biomet)	None	None
Linnanmaki^24^	2	Arthrex	Yes (Local Hospital District)	None	None	None
Thanasas^42^	1	Biomet Biologics	None	None	None	None
Gautam^10^	2	None listed	None	None	None	None
Martin^25^	1	None listed	Yes (FEDER Funds)	None	None	None
Gupta^13^	1	None listed	None	None	None	None
Krogh^18^	1	Recover GPS II (Biomet Biologic)	None	None	None	None
Peerbooms JC^33^	1	Biomet	Yes (Biomet, Dordrecht, the Netherlands)	None	None	None
Saglam^37^	1	Biostems, South Korea	Yes (Intraline Co., Ltd.)	None	None	None

*PRP*, platelet-rich plasma; *COI*, conflict of interest; *CMS*, Centers for Medicare and Medicaid Services; *AAOS*, American Academy of Orthopaedic Surgeons.

In total, 2 industry-affiliated studies (40%) had favorable outcomes for 6-month VAS, 1 (33%) at 12-month VAS, 1 (25%) at 6-month DASH, 1 (33%) at 12-month DASH, 0 (0%) at 6-month PRTEE, and 0 (0%) at 12-month PRTEE. For nonaffiliated studies, 5 nonaffiliated studies (55.6%) had favorable outcomes for 6-month VAS, 3 (50%) at 12-month VAS, 3 (42.8%) at 6-month DASH, 3 (60%) at 12-month DASH, 3 (60%) at 6-month PRTEE, and 1 (100%) at 12-month PRTEE.

Using a statistical significance level of 0.05, none of the timepoints were statistically significant when comparing outcomes of nonaffiliated studies to those of industry-affiliated studies. The *P* values for 6-month VAS, 12-month VAS, 6-month DASH, 12-month DASH, 6-month PRTEE, and 12-month PRTEE were 0.577, 0.635, 0.554, 0.465, 0.273, 0.157, respectively (Table 3).

**Table III:** Outcome scores.

Outcome	Affiliated, n	Nonaffiliated, n	*P* value
6 Mo VAS (n = 14)			.577
Favorable	2	5	
Analogous	3	4	
12 Mo VAS (n = 9)			.635
Favorable	1	3	
Analogous	2	3	
6 Mo DASH (n = 11)			.554
Favorable	1	3	
Analogous	3	4	
12 Mo DASH (n = 8)			.465
Favorable	1	3	
Analogous	2	2	
6 Mo PRTEE (n = 6)			.273
Favorable	0	3	
Analogous	1	2	
12 Mo PRTEE (n = 2)			.157
Favorable	0	1	
nalogous	1	0	

*VAS*, visual analog scale; *DASH*, disabilities of the arm, shoulder, and hand; *PRTEE*, patient-related tennis elbow evaluation.

## Discussion

The results of this systematic review study fail to demonstrate a statistically significant association between quantitative conclusions and industry affiliation when assessing the effectiveness of PRP treatment for lateral epicondylitis. Individual PROM outcomes, VAS and DASH, were not proven to be affected by presence of industry affiliation.

Included studies compared the treatment efficacy of lateral epicondylitis with PRP compared to corticosteroids at various timepoints. These studies demonstrated varied results at different timepoints. For example, a study by Gupta et al^13^ demonstrated that corticosteroids are better for treatment of lateral epicondylitis (based on VAS and DASH scores) in a short term (6 weeks); however, PRP is more effective in the long term (3 months and 1 year). Other studies such as the one by Linnanmäki et al,^24^ did not find PRP to be more effective than corticosteroids at any of their 4, 8, 12, 26, and 52-week timeframes. Such heterogeneity in study results was present throughout most of the included studies.

There is an increasing prevalence of explicit funding and industry affiliation leading to COI in medical literature.^8^^,^^6^^,^^45^ Industry affiliation in the orthopedic literature has been previously studied in order to determine whether the presence of affiliation is associated with reporting positive study results. Some prior studies have demonstrated an association between explicit funding and reporting of favorable outcomes with studied treatments. In our analysis, studies with industry affiliations were not significantly more likely to report the efficacy of PRP for treatment of lateral epicondylitis. For example, one included study by Mishra et al^27^ disclosed Biomet as an explicit funder of their study and their results found PRP to be superior to corticosteroids at the 8-, 12-, and 24-week timepoints. However, another study by Montalvan et al^28^ that disclosed Arthrex as an explicit funder of their study found that PRP was analogous to corticosteroids at their 1-, 3-, 6-, and 12-month timepoints. A study by Nesello et al,^29^ concluded that industry-sponsored studies investigating PRP therapy for various musculoskeletal disorders were more likely to report positive outcomes at a statistically significant level. They also concluded that the lower the quality of evidence, the more likely there were positive outcomes reported. Another key point in their study was that a large number of studies analyzed did not provide any information relating to financial sponsorship, which brings the frequency of articles sponsored by health industries into question.

Some previous studies have demonstrated an association between industry affiliation and the likelihood of reporting favorable study outcomes^34^^,^^1^^,^^30^^,^^7^^,^^22^^,^^21^^,^^6^^,^^45^; however, other studies have argued against this association.^41^^,^^43^^,^^26^^,^^23^^,^^4^ Similar to this study which did not produce findings that show an association, a recent systematic review by Canhnghi et al^41^ reported similar results. The authors of the study found that industry affiliation did not influence the results of level 1 and level 2 RCTs that investigated the treatment efficacy of PRP for knee osteoarthritis.^41^ Another study by Chou et al^4^ produced results similar to ours, in which they concluded that industry funding had no impact on the reporting of positive results for RCTs that investigated PRP in various musculoskeletal disorders (sprains, tendinopathies, arthritis, osteoporosis, etc.). However, they also concluded that included studies (41% of total studies included) that did not disclose sources of funding were more likely to have statistically significant findings.

There are several limitations to the study. Firstly, only studies that were submitted in English were considered. This may have led to relevant studies not being used in our analysis. The endpoints of the study were 6 months and 12 months for the VAS, DASH, and PRTEE. Other studies used endpoints ranging from 2 weeks to 2 years. Our endpoints were chosen to be 6 months and 12 months due to those being the most common reported time intervals in the RCT studies used in the meta-analysis. It is possible that the results could have been different over a longer timeframe. Another limitation was the low number of studies deemed to be industry-affiliated relative to the number of nonindustry-affiliated studies. Of the 26 studied level 1 and level 2 RCTs, only 6 were deemed to have industry affiliation either explicitly stated in the studied RCTs or reported on AAOS or CMS. This is due to the criteria set to declare a study as industry-affiliated. The criteria did not count explicitly stated funding or association with local hospitals or health ministry grants, but instead counted those with sponsorship from sources such as Arthrex, Stryker, DePuy, etc. Lastly, this systematic review does not contain a previous protocol, and it has not been registered in a database for systematic reviews.

The main strength of the study was the criteria we placed to consider an RCT as industry-affiliated. Many of the sources for this study were from authors located outside of the United States. The use of AAOS and CMS was key to investigate either author’s funding to assess for potential bias. CMS is predominantly used by US-based orthopedic surgeons, and despite the employment of CMS and AAOS, there is a possibility of having incorrectly declared a study as nonaffiliated, but AAOS is used by both US-based and international-based orthopedic surgeons. This allowed our study to better determine industry affiliation and reduce the chances of incorrectly declaring a study as nonindustry-affiliated.

## Conclusion

This study found that industry affiliation was not significantly associated with reporting a positive finding in studies investigating the use of PRP in lateral epicondylitis. While this study did not demonstrate an association between industry affiliation and PROM for treatment of lateral epicondylitis, the lack of consensus within literature, physicians should be cognizant of potential bias when using literature to make patient care recommendations. Other studies could also evaluate different endpoints to determine how industry affiliation impacts PROMs beyond 12 months. This same framework could be potentially used to investigate industry affiliation in treatment recommendations for other orthopedic pathologies.

## Disclaimers:

Funding: No funding was disclosed by the authors.

Conflicts of interest: The authors, their immediate families, and any research foundations with which they are affiliated have not received any financial payments or other benefits from any commercial entity related to the subject of this article.
